# Whole-Grain Highland Barley Attenuates Atherosclerosis Associated with NLRP3 Inflammasome Pathway and Gut Microbiota in ApoE^−/−^ Mice

**DOI:** 10.3390/nu15194186

**Published:** 2023-09-28

**Authors:** Tong Wu, Qinye Yu, Yingting Luo, Zijian Dai, Yuhong Zhang, Chao Wang, Qun Shen, Yong Xue

**Affiliations:** 1 National Engineering and Technology Research Center for Fruits and Vegetables, College of Food Science and Nutritional Engineering, China Agricultural University, Beijing 100083, China; estela_tong@163.com (T.W.); m13655709970@163.com (Q.Y.); s20213061018@cau.edu.cn (Y.L.); daizijian666@163.com (Z.D.); iamllacc1999@126.com (C.W.); 2 National Center of Technology Innovation (Deep Processing of Highland Barley) in Food Industry, Beijing 100083, China; 3 Tibet Academy of Agriculture and Animal Husbandry Sciences, Lhasa 860000, China; 18681357759@163.com

**Keywords:** highland barley, ApoE^−/−^ mice, atherosclerosis, NLRP3 inflammasome pathway, gut microbiota

## Abstract

The efficacy and mechanism of highland barley in the treatment of atherosclerosis have received little attention. Herein, we aimed to explore whether highland barley supplementation can prevent atherosclerosis progression and improve gut microbiota disorder in apolipoprotein E knockout (ApoE^−/−^) mice. Male ApoE^−/−^ mice were fed a high-fat diet with whole-grain highland barley (WHB) or refined highland barley for 18 weeks. WHB substantially inhibited the formation of atherosclerotic plaques, reduced serum tumor necrosis factor-α, and downregulated the expression of NLRP3 in the aorta. Furthermore, the 16S rRNA analysis revealed that highland barley supplementation helped to restore the dysregulation of the gut microbiota, as evidenced by an increase in the relative abundance of specific beneficial bacteria known for their anti-inflammatory properties, such as *Lachnospiraceae*, *Lactobacillus*, *Muribaculaceae*, and *Bifidobacterium*. Highland barley supplementation might alleviate atherosclerotic plaque formation by modulating the NLRP3 inflammasome pathway and the synthesis of anti-inflammatory metabolites by the gut microbiota.

## 1. Introduction

Cardiovascular diseases hold the highest mortality rate on a global scale based on findings in the global burden of disease study, and 85.1% of deaths were attributed to ischemic heart disease and stroke, which are closely related to atherosclerosis [1]. Similarly, the prevalence of cardiovascular diseases has increased rapidly in China and the number of patients is approaching 290 million now, accounting for more than 40% of all deaths in the population [2]. Atherosclerosis is a chronic inflammatory condition characterized by the accumulation of fatty substances, cholesterol, cellular waste, and calcium deposits on the inner walls of arteries. This accumulation leads to the formation of plaques, which can gradually narrow and harden the arteries, restricting blood flow to vital organs and tissues. Given that atherosclerosis serves as the primary cause of cardiovascular diseases, the prevention of atherosclerosis emerges as a critical issue that requires immediate attention, particularly in the context of the global scenario.

An unhealthy diet, particularly a high-fat diet, can cause anomalies in the cholesterol metabolism of the body. These anomalies contribute to the progression of cardiovascular diseases. Conversely, a healthy diet, such as whole-grain consumption, can prevent cardiovascular diseases [3]. Epidemiological research has shown a negative association between whole-grain consumption and the risk of cardiovascular diseases, as well as experiencing mortality from all causes [4]. Compared with refined grains, whole grains possess a higher concentration of phytosterols and soluble dietary fibers which could inhibit cholesterol absorption, regulate gut microbiota, and influence metabolism in the gut [5]. Highland barley, also known as naked barley and rice barley, is an annual herb in the genus Barley of the Gramineae family, with a nutritional structure of high dietary fiber (such as β-glucan), polyphenols, and other active ingredients [6]. Previous animal studies showed that highland barley had anti-obesity, anti-inflammatory, lipid-lowering, and glucose-lowering properties [7,8]. However, whether highland barley could contribute to the improvement of atherosclerosis is still unclear. Previous studies have found that whole-grain oats can alleviate atherosclerosis, likely due to improvements in lipid metabolism or the inflammatory state [9]. Considering that highland barley has both of these health benefits, it is reasonable to speculate that it may help improve atherosclerosis.

Whole grains are less processed compared to refined grains, and as a result, they retain a significant amount of dietary fiber such as pentosan, β-glucan, cellulose, and other bioactive compounds. These components cannot be directly digested in the small intestine and instead reach the colon, where they play a critical role in supporting gut health by modulating the composition and metabolic effects of the gut microbiota [10]. The gut microbiota ferments the dietary fiber from whole grains, producing short-chain fatty acids (SCFAs). The SCFAs not only help reduce inflammation in the host but also serve as an energy source for the cells in the colon [11]. This fermentation process and the subsequent production of SCFAs contribute to maintaining a healthy gut environment. Furthermore, the metabolites generated by the gut microbiota can enhance the completeness and functional effectiveness of the intestinal barrier, which prevents the penetration of harmful substances and pathogens such as bacterial lipopolysaccharide from crossing the intestinal lining into the bloodstream and surrounding tissues. This barrier function is crucial in reducing host inflammation and preventing the development of autoimmune disorders, allergies, and other health issues such as obesity and atherosclerosis [12]. However, the specific relationship between highland barley intervention and its impact on the gut microbiota composition in relation to atherosclerosis has not been thoroughly investigated. Prior research has demonstrated that anti-inflammatory strategies are able to decrease the incidence of cardiovascular events [13]. A growing body of evidence has indicated that nucleotide-binding oligomerization domain-like receptor protein 3 (NLRP3) inflammasomes are closely associated with the pathogenesis of atherosclerosis [14,15]. The classical NLRP3 inflammasome pathway is activated by two triggers, whose joint action drives apoptosis-associated speck-like protein (ASC) phosphorylation and produces ASC plaques to create atherosclerosis. The “first signal” is initiated by receptors such as TLR, leading to NF-κB activation and upregulation of NLRP3 and IL-1β. The “second signal” assembles the NLRP3/ASC/pro-caspase-1 complex, activating caspase-1, which releases IL-1β. IL-1β triggers inflammation and the release of other cytokines, such as IL-18, IL-6, and TNF-α, intensifying the inflammatory response [16,17]. One study examined the effect of dietary whole-grain buckwheat and oat and found that both could inhibit the increase in NLRP3 inflammasome in benzo[a]pyrene-induced mice [18]. Similarly, in mice fed a high-fat diet, fermented Tartary buckwheat has shown the potential to reduce body weight gain and address gut microbiota dysbiosis through the NLRP3/Caspase-1 signaling pathway [19]. Interestingly, butyrate fermented from dietary fiber by specific microbiota could stimulate signaling of GPR109A and GPR43, and then suppress NLRP3 inflammasome [20]. However, it remains unclear whether whole-grain highland barley could regulate the NLRP3 inflammasome pathway.

Considering the interconnection of gut microbiota, host inflammation, and atherosclerosis, we formulated a hypothesis that highland barley could attenuate atherosclerosis by modulating the NLRP3 inflammasome pathway through the regulation of gut microbiota. Then, we explored the effects of whole-grain highland barley (WHB) and refined highland barley (RHB) on atherosclerotic lesion development, the NLRP3 inflammasome pathway, and gut microbiota in male ApoE^−/−^ mice to confirm this hypothesis. Our results showed that WHB could attenuate atherosclerosis, which might be associated with the improvement of the NLRP3 inflammasome pathway and gut microbiota.

## 2. Materials and Methods

### 2.1. Animals

Thirty 7-week-old male ApoE^−/−^ mice were provided by Vital River Laboratory Animal Technology Co., Ltd. (Beijing, China). The ApoE^−/−^ mouse is a well-known animal model for studying the association of nutritional interventions with atherosclerosis [21,22]. The mice were housed in standard cages on a 12 h light-dark cycle, at a constant temperature (23 ± 2 °C), and with a relative humidity of 55 ± 5%. The mice had unrestricted access to both food and water throughout the duration of the experiment.

### 2.2. Diets and Experimental Design

The Tibet Academy of Agriculture and Animal Sciences provided highland barley (Zangqing 320) with different peeling degrees (at 0% or 64%). Highland barley grains were selected, followed by thorough washing and drying. Then, the grains were ground into a fine powder with a particle size of 60 mesh and stored at 4 °C.

After ten days of acclimatization, all ApoE^−/−^ mice were assigned to three groups randomly (*n* = 10 per group): (1) ApoE^−/−^ mice fed with a high-fat diet (HFD; 60 kcal% fat, D12492; Research Diets, New Brunswick, NJ, USA); (2) an HFD supplemented with 30% (*w*/*w*) WHB; and (3) an HFD supplemented with 30% (*w*/*w*) RHB. Under the guidance of HFD D12492, the nutritional compositions of all diets used in the study were established. For specific details regarding the energy densities and compositions of the experimental diets, please refer to Table S1. The highland barley supplementation level in the diet was determined based on previous studies conducted in this field [7,8]. Each group of mice in the study was fed for the same period of 18 weeks. Food intake and body weight were weighed and recorded once a week. Feces samples were taken from each mouse in the group in the 18th week, promptly immersed in liquid nitrogen, and then stored at −80 °C. At the end of the experiment, mice were administered anesthesia using chloral hydrate after an 8 h fasting period. After cervical dislocation, the livers and aortas were quickly harvested. Some were fixed in 4% paraformaldehyde solution, while the rest were stored at −80 °C.

### 2.3. Serum Lipids and Inflammatory Cytokines

The blood samples were collected in EP tubes from the internal canthal vein and centrifuged (4 °C, 3500 rpm, 15 min). The total cholesterol (TC), triglycerides (TG), low-density lipoprotein-cholesterol (LDL-C), and high-density lipoprotein-cholesterol (HDL-C) were detected using commercially available kits (Nanjing Jiancheng Bioengineering Institute, Nanjing, Jiangsu, China). The enzyme-linked immunosorbent assay (ELISA) was used to assess the levels of serum cytokines, including interleukin-1β (IL-1β) and tumor necrosis factor-α (TNF-α) (Jiangsu Meimian Industrial Co., Ltd., Yancheng, China).

### 2.4. Evaluation of Atherosclerotic Plaques

The aortas and hearts of the mice were harvested under a dissecting microscope, and any adventitial fat and surrounding tissues were gently removed. Half of the hearts (*n* = 5 per group, selected randomly) were fixed with 4% paraformaldehyde (*v*/*v*) before undergoing cryosectioning for oil-red staining. The tissue was dehydrated, dried, and embedded in optimal cutting temperature compounds. Subsequently, 8–10 μm thick sections were cut from the aortic sinus using a frozen microtome (CRYOSTAR NX50, Thermo, Waltham, MA, USA) and stained with oil red. The stained sections were sealed, and a microscope (BX51, Olympus Corporation, Tokyo, Japan) was used to observe them. The percentage of plaque area was evaluated and quantified using Image-Pro Plus 6.0 software. The aorta was also fixed with 4% paraformaldehyde (*v*/*v*) and decolored after oil-red staining for 4 h until the arterial wall appeared white. Then, the stained slices were sealed and observed under a microscope (BX51, Olympus Corporation, Tokyo, Japan). The percentage of plaque areas was estimated using Image-Pro Plus 6.0 software. In addition, the aorta was fixed with 4% paraformaldehyde (*v*/*v*) for more than 24 h, stained with oil red, and decolorized until the artery wall appeared white. Images were obtained, and the arterial gross wall and atherosclerotic plaque surface area were assessed using Image-Pro Plus 6.0 software.

### 2.5. Evaluation of Aortic Gene Expression

Q-PCR was used to evaluate the expression of the identified genes in the aorta (*n* = 5 per group) using a C1000 Touch thermocycler (Bio-Rad, Hercules, CA, USA). The eluted RNA was used in a reverse transcription procedure. In a 20 μL reaction at 42 °C for 1 h, RevertAid 100T was employed to convert 1 μg total RNA of each aorta into cDNA. For Q-PCR, the reverse transcription products and primer, as well as Bio-SYBR Rad’s Green I Master Mix, were employed in a 10 μL volume (Bio-Rad). The primer sequences and gene sizes were 5′-AAGATCAATGGCTACACAGG-3′ and 3′-CCTCAATGTCTTCTTTCTGC-5′ for NF-κB; 5′-TCTTCTCAAGTCTAAGCACCAAC-3′ and 3′-ACAGCAATCTGATTCCAAAGTC-5′ for NLRP3; 5′-CTTCAGGCAGGCAGTATCACTC-3′ and 3′-TTGTTGTTCATCTCGGAGCC-5′ for IL-1β; 5′-CCAACAAGGAGGAGAAGTTCC-3′ and 3′-CTCTGCTTGGTGGTTTGCTAC-5′ for TNF-α; 5′-GCATGGCCTTCCGTGTTC-3′ and 3′-GATGTCATCATACTTGGCAGGTTT-5′ for GAPDH. Q-PCR amplification was carried out as follows: 1 cycle of 95 °C for 3 min, followed by 39 cycles of 95 °C for 10 s, 60 °C for 30 s, and 65 °C for 5 s, respectively. Following amplification, a melt curve for the reaction product was created to provide evidence for a single reaction product. After checking the accuracy of denaturing agarose gel electrophoresis, all mRNA expression data were standardized to GAPDH mRNA expression in the same sample.

### 2.6. DNA Extraction of Gut Microbiota and 16S rRNA Gene Sequencing

The genomic DNA from fecal samples was extracted using the E.Z.N.A.^®^ soil DNA kit (Omega Bio-tek, Norcross, GA, USA). DNA sample quality was assessed by NanoDrop 2000 UV-vis spectrophotometer (Thermo Scientific, Wilmington, MA, USA) after being checked by agarose gel (1%). Afterward, the V3-V4 region was amplified in the ABI GeneAmp^®^ 9700 thermocycler (ABI, Foster City, CA, USA). Extraction, purification, and quantification of PCR products using agarose gel (2%), AxyPrep DNA Gel Extraction Kit (Axygen Biosciences, Union City, CA, USA), and Quantus Fluorometer (Promega, Madison, WI, USA) were performed. On the Illumina MiSeq PE300 platform or the Illumina NovaSeq PE250 platform (San Diego, CA, USA), amplicons were sequenced with paired ends. Sequence data analysis was conducted using UPARSE 7.1 software, where high-quality sequences with a similarity of at least 97% were clustered into operational taxonomic units (OTUs). A similarity of 70% was used for the taxonomic classification of sequences based on the 16S rRNA gene database [23].

The alpha-diversity index of bacterial communities at the OTU level was examined by the Wilcoxon rank-sum test. To analyze the beta diversity, nonmetric multidimensional scaling (NMDS) and principal coordinates analysis (PCoA) were conducted at the genus level using the Bray–Curtis distance. The most discriminant taxa between groups were analyzed using Linear discriminant analysis (LDA) of effect size (LEfSe), with an LDA score of ≥3.0.

### 2.7. Statistical Analysis

GraphPad Prism 8 (GraphPad Software Inc., San Diego, CA, USA) was utilized to perform all statistical analysis. One-way analysis of variance (ANOVA), followed by Tukey’s multiple comparison test, was used to assess the data. The results were presented as the mean ± standard error of the mean (SEM). Statistical significance was considered when *p* < 0.05.

## 3. Results

### 3.1. Body Weight, Liver Coefficient and Serum Lipids

**Figure 1:**
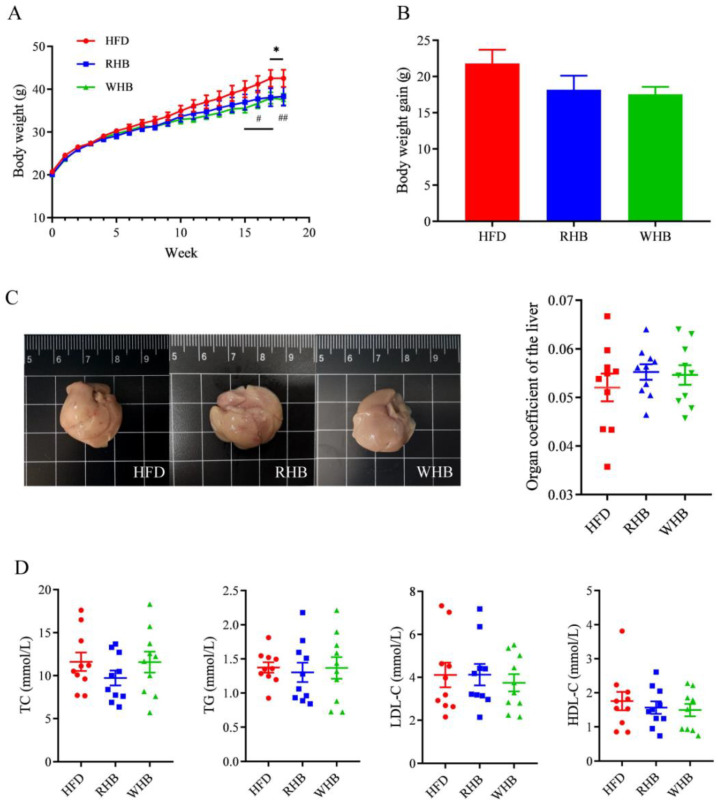
Effect of highland barley on the body weight, liver coefficient and serum lipids in ApoE^−/−^ mice. (**A**) Body weight. (**B**) Body weight gain. (**C**) Liver coefficient. (**D**) Serum lipids. Results are presented as mean ± SEM (*n* = 10). Differences were considered statistically significant by one-way ANOVA followed by Tukey’s multiple comparison. * *p* < 0.05, RHB group vs. HFD group; # *p* < 0.05, ## *p* < 0.01, WHB group vs. HFD group. HFD, the group fed a high-fat diet; RHB, the group fed HFD containing 30% refined highland barley; WHB, the group fed HFD containing 30% whole-grain highland barley; TC, total cholesterol; TG, triglycerides; LDL-C, low-density lipoprotein-cholesterol; HDL-C, high-density lipoprotein-cholesterol.

Body weight changes are shown in Figure 1. Over a period of 18 weeks, there was no significant difference in body weight among the three groups during the initial 15 weeks. After the intervention with RHB and WHB, body weight significantly decreased at 17 and 15 weeks, respectively, compared to the HFD group. This difference between the WHB group and the HFD group became even more significant by the 18th week. However, no difference was considered significant in weight between the two intervention groups (Figure 1A). Similarly, there was a downward trend of body weight gain with highland barley supplementation after the 18-week administration (Figure 1B). In addition, there was no notable change in the liver’s organ coefficient observed among the HFD, RHB, and WHB groups (Figure 1C), which indicates that HB does not have a toxic effect on the body. Meanwhile, the concentrations of serum TC, TG, HDL-C, and LDL-C were similar among the three groups (Figure 1D). These data implicated that the supplementation of HB could suppress body weight increase, with whole-grain highland barley having a stronger benefit.

### 3.2. Atherosclerotic Plaques, Aortic Gene Expression and Serum Inflammatory Cytokines

**Figure 2:**
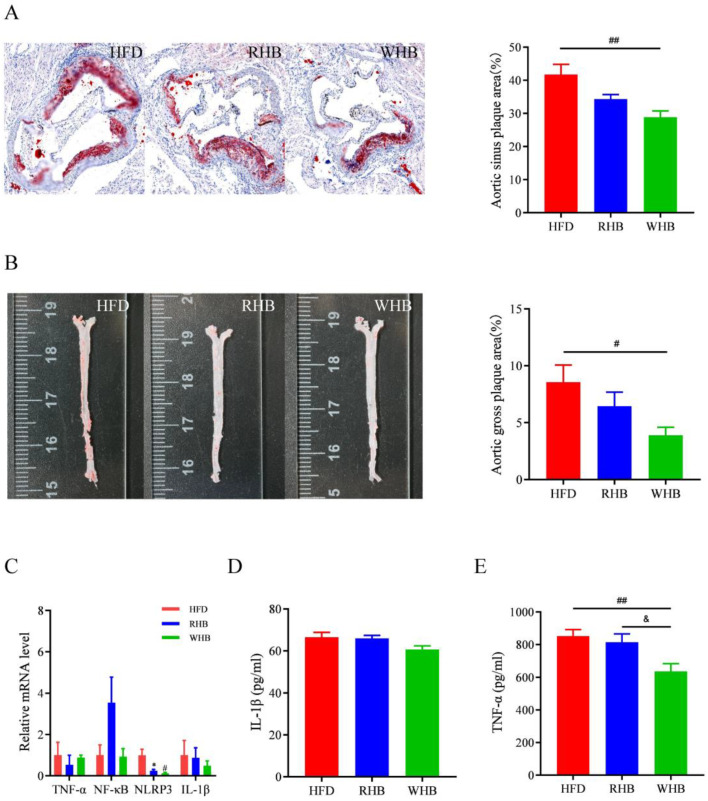
Effect of highland barley supplementation on the atherosclerotic plaques, aortic gene expression and serum inflammatory cytokines in ApoE^−/−^ mice. (**A**) Aortic sinus plaques (*n* = 5). (**B**) Gross plaque image (*n* = 5). (**C**) Aortic gene expression (*n* = 3). (**D**) Serum IL-1β levels (*n* = 10). (**E**) Serum TNF-α levels (*n* = 10). Results are presented as mean ± SEM. Differences were considered statistically significant by one-way ANOVA followed by Tukey’s multiple comparison. * *p* < 0.05, RHB group vs. HFD group; # *p* < 0.05, ## *p* < 0.01, WHB group vs. HFD group; & *p* < 0.05, RHB group vs. WHB group. HFD, the group fed a high-fat diet; RHB, the group fed HFD containing 30% refined highland barley; WHB, the group fed HFD containing 30% whole-grain highland barley.

In the study, we examined atherosclerotic plaques in ApoE^−/−^ mice and observed their presence in both the aortic sinus and gross plaque images. Specifically, in the area of the aortic sinus, the group fed an HFD exhibited a substantial number of lipid deposits in the intima of the aorta. These deposits formed atheromatous plaques that protruded significantly into the lumen of the aorta. In contrast, the group fed RHB had a smaller number of lipid deposits in the intima of the aorta, with limited formation of atheromatous plaques in the aortic wall. Furthermore, we observed that the group fed WHB had significantly fewer atherosclerotic plaques compared to the HFD group (Figure 2A, *p* < 0.05). In addition, the aortic walls of the HFD group had a large number of red-stained lipid plaque masses, although there was no notable change among the three groups. In contrast, the aortic walls in the RHB and WHB groups were largely white, with no red-stained lipid plaque masses (Figure 2B). These observations indicate that both RHB and WHB demonstrated properties that could help mitigate the progression of atherosclerotic plaques in the arteries. However, WHB appears to be more effective in reducing the buildup of atherosclerotic plaques compared to RHB.

As TNF-α, NF-κB, NLRP3, and IL-1β are crucial genes in the formation of ASC plaques to formulate atherosclerosis, we evaluated their expression in the aorta by Q-PCR. The RHB and WHB group exhibited significantly lower levels of NLRP3 when compared with the HFD group. Additionally, there was a downward trend in the IL-1β level with highland barley supplementation (Figure 2C). Furthermore, the serum level of IL-1β also went down after highland barley supplementation (Figure 2D). The serum level of TNF-α significantly reduced in both the RHB and WHB groups compared with the HFD group (Figure 2E, both *p* < 0.05).

### 3.3. Effects of HB Supplementation on HFD-Induced Alterations in Gut Microbiota Structure

**Figure 3:**
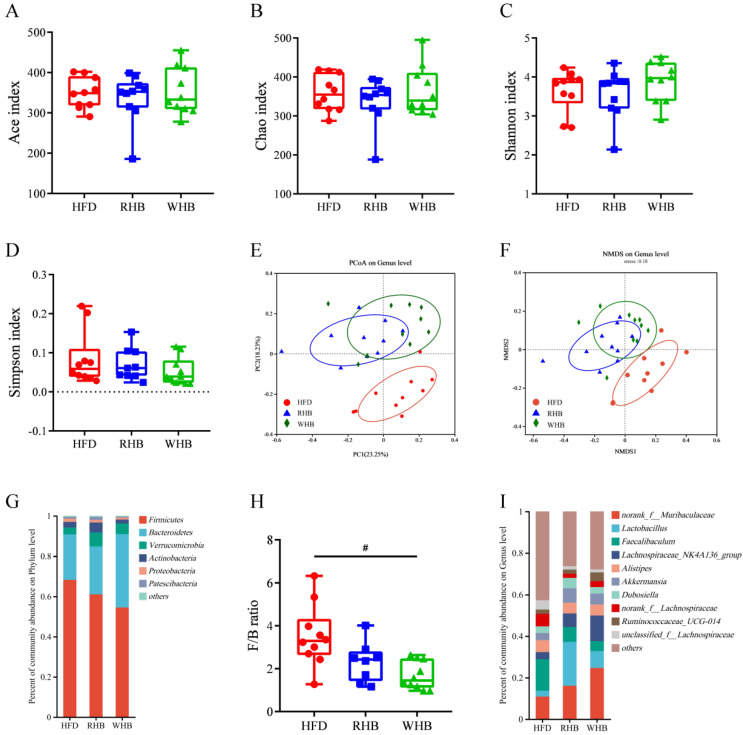
Effect of HB on the diversity of gut microbiota in HFD mice. (**A**–**D**) Changes in Ace (**A**), Chao (**B**), Shannon (**C**), and Simpson (**D**) of alpha diversity index. (**E**) Principal coordinate analysis (PCoA) and (**F**) Nonmetric multidimensional scaling (NMDS) on genus level based on Bray–Curtis dissimilarity. (**G**) Bacterial spectrum at the phylum level. (**H**) Firmicutes/Bacteroidetes (F/B) ratio. Results are presented as mean ± SEM (*n* = 10). (**I**) Bacterial spectrum at the genus level. Differences were considered statistically significant by one-way ANOVA followed by Tukey’s multiple comparison. # *p* < 0.05, HFD group vs. WHB group. HFD, the group fed a high-fat diet; RHB, the group fed HFD containing 30% refined highland barley; WHB, the group fed HFD containing 30% whole-grain highland barley.

To gain a deeper understanding of how highland barley supplementation affects the dysbiosis of gut microbiota caused by an HFD, 16S rRNA gene sequencing was employed to identify and analyze differences in the structure of gut microbiota. There was a total of 1,550,282 high-quality sequencing reads yielded by 30 fecal samples. Alpha diversity analysis at the OTU level showed no significant differences between the three groups (Figure 3A–D). The beta diversity of the gut microbiota was assessed by the Bray–Curtis-based PCoA and NMDS. The RHB and WHB groups were segregated from the HFD group. The fact that the microbiota profile of the groups was clearly clustered showed that HB supplementation might reverse the HFD-induced alterations in gut microbiota composition. Despite their separation, there was a great deal of overlap between the two intervention groups, which could be due to the similarity in nutritional composition between WHB and RHB. (Figure 3E,F). We further observed the bacterial spectrum at the phylum and genus levels to fully comprehend the impact of highland barley. The results showed that phylum *Firmicutes*, *Bacteroidetes*, *Verrucomicrobia*, and *Actinobacteria* dominated the gut microbiota (Figure 3G). The intervention of RHB and WHB decreased the relative abundance of *Firmicutes*, while *Bacteroidetes* was increased in the WHB group when compared to the HFD group. Notably, the intervention of both RHB and WHB reduced the increase in the *Firmicutes*/*Bacteroidetes* (F/B) ratio induced by HFD. WHB had a considerably stronger inhibitory impact (Figure 3H). At the genus level, *norank_f_Muribaculaceae*, *Lactobacillus*, *Lachnospiraceae_NK4A136,* and *Akkermansia* abundances were higher in the RHB and WHB groups than in the HFD group (Figure 3I).

**Figure 4:**
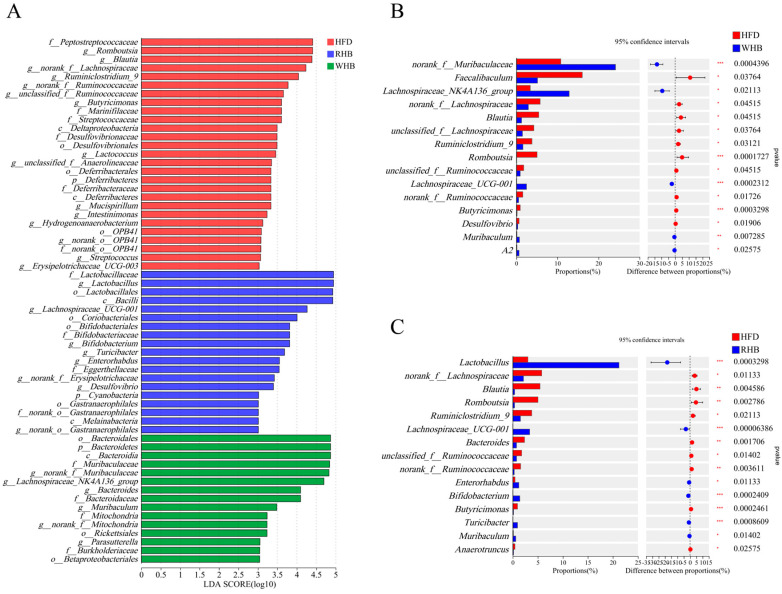
Effect of highland barley on the composition of gut microbiota in HFD mice. (**A**,**B**) Mean proportions of 15 significantly different genera in mice between RHB, WHB, and HFD groups (Welch’s *t*-test was performed to assess the difference between the two groups). (**C**) Linear discriminant analysis (LDA ≥ 3.0) scores derived from LEfSe analysis. * *p* < 0.05, ** *p* < 0.01, *** *p* < 0.001. HFD, the group fed a high-fat diet; RHB, the group fed HFD containing 30% refined highland barley; WHB, the group fed HFD containing 30% whole-grain highland barley.

In order to identify the specific microbial taxa from phylum to genus that contributed significantly to the differences observed with highland barley intervention, a microbial community analysis using LEfSe was conducted. The HFD group exhibited a predominance of *Romboutsia*, *unclassified_f_Anaerolineaceae*, *Mucispirillum*, and *Streptococcus* at the genus level, implying that their abundance increased following HFD. In contrast, supplementation with HB not only preserved the situation but also increased the abundance of certain beneficial microbiota. The RHB featured the genera *Lactobacillus*, *Lachnospiraceae_UCG-001,* and *Bifidobacterium*, while the WHB group featured the genera *norank_f_Muribaculaceae*, *Lachnospiraceae_NK4A136*, *Bacteroides* and *Muribaculum*, suggesting that their abundance was promoted through highland barley intervention (Figure 4A). In the meantime, we compared the variations among the three groups at the genus level through Wilcoxon rank-sum. Among the top 15 genera, highland barley intervention led to an increase in *Lachnospiraceae_UCG-001* and *Muribaculum*. Additionally, an increased abundance of *Lactobacillus* and *Bifidobacterium* was observed in the RHB group, while an increased abundance of *norank_f_Muribaculaceae* and *Lachnospiraceae_NK4A136* was observed in the WHB group (Figure 4B,C).

**Figure 5:**
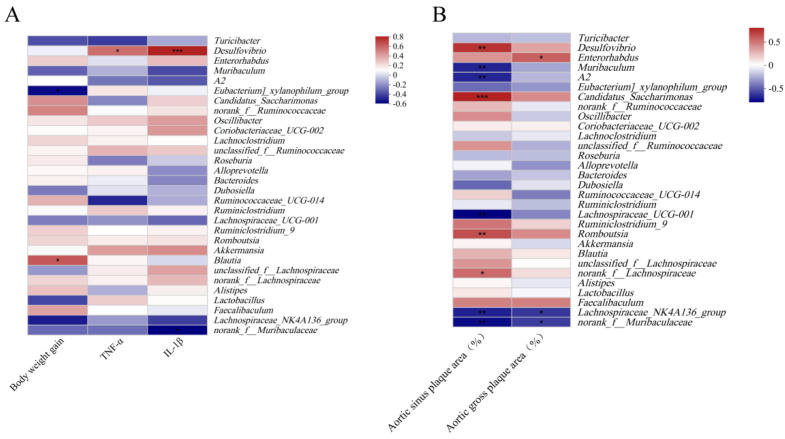
Correlation between physiological indicators and gut microbiota. (**A**) Spearman correlation heatmap between gut microbiota and body weight gain and serum inflammatory cytokine levels. (**B**) Spearman correlation heatmap between gut microbiota and atherosclerotic plaques. * *p* < 0.05, ** *p* < 0.01, *** *p* < 0.001.

We further explored the correlation of the top 30 genera with physiological indicators. The abundance of *norank_f_Muribaculaceae*, *Lachnospiraceae_NK4A136*, *Muribaculum*, and *Lachnospiraceae_UCG-001* was negatively correlated with body weight gain, serum inflammatory cytokine levels, and aortic plaque areas. In contrast, the abundance of *Romboutsia* and *Desulfovibrio* was positively correlated with inflammatory cytokine levels and aortic plaque areas (Figure 5).

## 4. Discussion

Numerous human studies revealed that there is a negative association between the risk of atherosclerosis and the consumption of whole grains. This association can largely be attributed to the benefits of whole grains in improving lipid metabolism and reducing inflammation within the body. Previous animal studies have also indicated that highland barley possesses properties that can combat obesity and inflammation, and lower lipid levels. However, it remains uncertain whether whole-grain highland barley can effectively attenuate atherosclerosis. In this research, we aimed to explore the influences of whole-grain highland barley on ameliorating atherosclerosis in ApoE^−/−^ mice fed an HFD. Additionally, we sought to explore the underlying mechanisms of aortic inflammation and gut microbiota. Our results revealed that the intervention with whole-grain highland barley effectively suppressed HFD-induced weight gain and atherosclerotic plaque formation. Moreover, it significantly downregulated the expression of NLRP3 in the aorta and reduced serum levels of inflammatory factors. Furthermore, the highland barley intervention contributed to the restoration of dysregulated gut microbiota and a significant increase in the relative abundance of specific beneficial bacteria associated with the suppression of host inflammation. These findings suggest that whole-grain highland barley has the potential to effectively attenuate atherosclerosis, and highlight the importance of maintaining a healthy gut microbiota as a potential therapeutic approach to regulate inflammation and promote cardiovascular well-being.

Although our study did not show significant hypoglycemic effects of HB in ApoE^−/−^ mice fed an HFD, it is plausible that HB can still attenuate atherosclerosis by reducing inflammation. There is a mounting body of evidence indicating that gut microbiota acts as an essential regulator between diets and the host [10]. Dietary intervention with diet fiber may impact the overall gut microbiota composition, preventing inflammatory effects and metabolic abnormalities. The F/B ratio has been studied and linked to the morbidity of illness [24]. Studies have shown that obese children tend to exhibit higher F/B ratios compared to healthy children [25]. In our study, supplementation with highland barley effectively reversed the increase in the F/B ratio in mice fed an HFD. Furthermore, the presence of specific bacterial species has been closely associated with particular biological effects. Consistent with findings from other animal experiments involving highland barley [8,26], we observed that highland barley supplementation led to alterations in the composition of the intestinal microbiota, including an increase in beneficial bacteria such as *Lachnospiraceae*, *Lactobacillus*, *Muribaculaceae*, and *Bifidobacterium*. These bacteria are known to have the ability to degrade mucus, thereby competing with pathogens and serving as ecological guardians in maintaining gut barrier integrity [27]. *Lachnospiraceae* could display healthy effects by producing SCFAs and converting primary bile acids to secondary bile acids [28]. *Lactobacillus* could reduce dietary triglyceride absorption and serum levels of pro-inflammatory markers [29]. *Bifidobacterium* is an anaerobic beneficial bacterium that could foster the production of SCFAs and lead to anti-inflammatory effects [30]. Given the potential of these gut microbes to suppress inflammation, which is a crucial factor in the process of atherosclerosis, it is reasonable to associate the beneficial effects of highland barley with its regulation of host microbes.

Both experimental and clinical studies have provided compelling evidence that highlights the crucial role of inflammation in the development of atherosclerosis [31]. The immune system could trigger a series of persistent inflammatory responses in the stages of atherosclerosis development. NLRP3 inflammasome vesicles are the most well-studied as introduced previously. The classical NLRP3 inflammasome pathway is activated by two triggers, whose joint action drives ASC phosphorylation and produces ASC plaques to create atherosclerosis. The “first signal” is mediated by TLR or other pattern recognition receptors, which activates the NF-κB pathway and upregulates the expression of NLRP3 and the downstream precursor IL-1β. The “second signal” is responsible for assembling the NLRP3/ASC/pro-caspase-1 protein complex, in which pro-caspase-1 is self-sheared into an active form. The activated caspase-1 then releases the IL-1β precursor into the extracellular compartment [16]. IL-1β, as a potent inducer of inflammation, promotes the release of other pro-inflammatory cytokines such as IL-18, IL-6, and TNF-α through the IL-1β receptor signaling pathway, thereby exacerbating the inflammatory response [17]. Current studies have revealed the ability of HB to lower the risk of cardiovascular disease via anti-inflammation. Here, we observed that the level of aortic inflammatory factor significantly down-regulated in the WHB and RHB group compared to the HFD group. In accordance with other vitro and vivo studies, the serum level of IL-1β and TNF-α comes down after being supplemented with HB [32,33]. From these findings, it could be inferred that the NLRP3 inflammasome pathway is crucial in the development of atherosclerosis and that HB is beneficial in reducing the inflammatory response of the host.

Consumption of whole-grain barley may offer a wealth of nutritional benefits for the host. Whole-grain barley is rich in β-glucans, vitamin E, and various classes of phenolic compounds [6]. These bioactive compounds have the ability to stimulate the growth of beneficial bacteria and regulate metabolic effects in the gut. The intestinal microbiota could ferment β-glucan, and facilitate its digestion and absorption. This process leads to the production of active metabolites, including SCFAs, known for their pharmacological effects. Previous studies have indicated that SCFAs help maintain the activity of mucosal immune cells, preserve the integrity of the intestinal lining, reduce colon pH to inhibit harmful bacterial growth, and regulate host inflammation [11]. Vitamin E, specifically α-tocopherol, is widely acknowledged as an important fat-soluble antioxidant. The applications of vitamin E hold significant importance in both the prevention and complementary treatment of atherosclerosis [34]. Recent research conducted on rats supports the notion that intestinal microbiota plays a significant role in degrading various forms of vitamin E and increasing their bioavailability [35]. Furthermore, a significant portion (70–95%) of phenolic compounds in whole-grain cereals is either bound within cell wall polysaccharides or trapped within cells [36]. However, these phenolics can become bio-accessible in the colon through microbial fermentation, producing various metabolic products including phenolic acids and phenolic acid-SCFA conjugates. These compounds possess the ability to inhibit the promotion or activation of pro-inflammatory mediators, thereby exhibiting anti-inflammatory properties in the host. Hence, it is reasonable to interpret that anti-inflammatory metabolites produced through microbial fermentation contribute to the suppression of atherosclerosis. And in our study, the abundance of microbiota enriched by HB supplementation such as *norank_f_Muribaculaceae*, *Lachnospiraceae_NK4A136*, *Muribaculum,* and *Lachnospiraceae_UCG-001* were negatively correlated with serum inflammatory cytokine levels and aortic plaque areas. Based on the fact that whole-grain cereals undergo less processing and maintain a higher content of bioactive compounds, it has been observed that WHB is more effective in preventing fat accumulation and reducing the area of atherosclerotic plaque compared to RHB.

As far as we know, this study is the first to study the effect of highland barley on atherosclerotic plaques by evaluating aortic pathology. However, several limitations should be considered. The study was carried out at the food level, thus the possible effects of certain bioactive components such as phenolic compounds and β-glucans were not defined. In addition, although the above data indicated that highland-barley-induced gut microbial regulations were effective in ameliorating atherosclerosis, the causal association between them has not yet been established. Furthermore, we observed that the biological activities of RHB are weaker than those of WHB, suggesting the essential part HB bran plays in the process. These findings and challenges open up a remarkable way for further study of the nutritional function of highland barley.

## 5. Conclusions

In this study, we aimed to examine the potential of highland barley in mitigating atherosclerosis by investigating its effects on the NLRP3 inflammasome pathway and gut microbiota. The results of our study provide support for this hypothesis, as we found that WHB exhibits stronger biological activities compared to refined highland barley RHB. One of the key factors contributing to the enhanced biological activities of WHB is its minimal processing, which allows it to retain a substantial amount of bioactive compounds. These compounds play a critical role in stimulating the growth and regulating the metabolic effects of the microbiota in the large intestine. As a result, WHB demonstrates an anti-inflammatory capacity, which is particularly relevant to the development of atherosclerosis.

## Data Availability

Data presented in this study are available on request from the corresponding author.

## Supplementary Materials

The following supporting information can be downloaded at: https://www.mdpi.com/article/10.3390/nu15194186/s1, Table S1: Specific formulas of different feeds.
