# Supplementary material for: Whole-Grain Highland Barley Attenuates Atherosclerosis Associated with NLRP3 Inflammasome Pathway and Gut Microbiota in ApoE−/− Mice
Source: Nutrients. 2023 Sep 28;15(19):4186. doi: 10.3390/nu15194186 (PMC10574078; doi:10.3390/nu15194186)
Supplement: Supplementary file 1 [file nutrients-15-04186-s001.zip › nutrients-2635207-supplementary.pdf]

Table S1. Specific formulas of different feeds.

Ingredient (g/kg)	HFD	WHB	RHB
WHB flour	-	300	-
RHB flour	-	-	300
Casein, 80 Mesh	258.45	223.85	225.15
L-Cystine	3.88	3.88	3.88
Maltodextrin 10	161.53	0	0
Sucrose	88.91	1.34	1.7
Cellulose, BW200	64.61	56.62	55.08
Soybean Oil	32.31	25	24.28
Lard	316.6	316.6	316.6
Mineral Mix, S10026	12.92	12.92	12.92
DiCalcium Phosphate	16.8	16.8	16.8
Calcium Carbonate	7.11	7.11	7.11
Potassium Citrate, 1 H_2O	21.32	21.32	21.32
Vitamin Mix, V10001	12.92	12.92	12.92
Choline Bitartrate	2.58	2.58	2.58
FD&C Blue Dye #1	0.06	0	0
Total (g)	1000.00	1000.34	1000.94

RHB, refined highland barley; WHB, whole-grain highland barley.
